# Increased Vesicular Monoamine Transporter 2 (VMAT2) and Dopamine Transporter (DAT) Expression in Adolescent Brain Development: A Longitudinal Micro-PET/CT Study in Rodent

**DOI:** 10.3389/fnins.2018.01052

**Published:** 2019-01-15

**Authors:** Donglang Jiang, Xiuhong Lu, Zijing Li, Nicklas Rydberg, Chuantao Zuo, Fangyu Peng, Fengchun Hua, Yihui Guan, Fang Xie

**Affiliations:** ^1^PET Center, Huashan Hospital, Fudan University, Shanghai, China; ^2^Center for Molecular Imaging and Translational Medicine, State Key Laboratory of Molecular Vaccinology and Molecular Diagnostics, School of Public Health, Xiamen University, Xiamen, China; ^3^Department of Radiology, The University of Texas Southwestern Medical Center, Dallas, TX, United States

**Keywords:** positron emission tomography, brain development, vesicular monoamine transporter 2 (VMAT2), dopamine transporter (DAT), glucose metabolism

## Abstract

**Background:** Brain development and maturation in adolescence is a complex process with active changes of metabolic and neurotransmission pathways. Positron emission tomography (PET) is a useful imaging modality for tracking metabolic and functional changes in adolescent brain. In this study, changes of glucose metabolism, expression of vesicular monoamine transporter 2 and dopamine transporter during adolescent brain development in rats were investigated with PET/CT.

**Methods:** A longitudinal PET/CT study of age-dependent changes of VMAT2, DAT and glucose metabolism in adolescent brain was conducted in a group of Wistar rats (*n* = 6) post sequential intravenous injection of ^18^F-PF-(+)-DTBZ, ^11^C-CFT, and ^18^F-FDG, respectively. PET acquisition was performed at 2, 4, 9, and 12 months of age. Radiotracer uptake in different brain regions, including the striatum, cerebellum, and hippocampus, were quantified and recorded as Standardized uptake value (SUV) and striatal specific uptake ratio (SUVR: SUV in brain regions/SUV in cerebellum).

**Results:** Variable uptake of ^18^F-PF-(+)-DTBZ and ^11^C-CFT were detected, with highest level uptake in the striatum and accumbens. There was significant age-dependent increase of ^18^F-PF-(+)-DTBZ and ^11^C-CFT uptake in the striatum from 2 months of age (SUV: 1.36 ± 0.22, 1.37 ± 0.39, respectively), to 4 months (SUV: 2.22 ± 0.29, 2.04 ± 0.33), 9 months (1.98 ± 0.34, 2.09 ± 0.18), 12 months (SUV: 1.93 ± 0.19, 2.00 ± 0.17) of age, SUV of ^18^F-FDG also increased from 2 months of age to older ages (SUV in the striatum: 3.71 ± 0.78 at 2 month, 5.28 ± 0.81, 5.14 ± 0.73, 4.94 ± 0.50 at 4, 9, 12 month, respectively).

**Conclusion:** Age-dependent increases of striatal of ^18^F-FDG, ^18^F-PF-(+)-DTBZ, and ^11^C-CFT uptake were detected in rats from 2 to 4 month of age, demonstrating striatal development presents over the first 4 months of age. Four months of age can be considered a safe threshold to launch brain disease studies for exclusion of confusion of continuing tissue development. These findings support further investigation of age-dependent changes in expression of DAT, VMAT2, and glucose metabolism for their potential use as a new imaging biomarker for study of brain development and functional maturation.

## Introduction

Brain development is a dynamic and complex process, including the change of brain volume and morphology, metabolism, expression of receptors and transporters, and function (Chugani and Phelps, 1986; Chugani, 1998; Khundrakpam et al., 2016). A number of investigations indicated brain regional growth with changes of cortical thickness during brain development and maturation using MR (Sowell et al., 2004; Aljabar et al., 2008; Mengler et al., 2014). However, metabolic and functional changes associated with brain development and maturation remain largely unknown or poorly understood, particularly in adolescent population. Because of vulnerability caused by un-mature status of brain development, the adolescent population is at a high risk for development of various mental health problems, such as uncontrolled violence, depression, and drug abuse. It is important to explore metabolic changes and changes of receptors or neurotransmission pathways during brain development and maturation process in adolescent population.

Positron emission tomography (PET) is a particularly useful functional imaging tool for tracking metabolic changes or changes of neuro-receptor expression during brain development using a radiotracer. PET imaging with [^18^F]fluoro-2-deoxy-D-glucose (^18^F-FDG) is widely used to detect alterations of cerebral glucose metabolism in brain development, aging, and various brain disorders (Mosconi et al., 2008; Smailagic et al., 2015). Some study present the glucose metabolism change in brain maturation using ^18^F-FDG/PET (Choi et al., 2015). Using radioligands binding to brain receptors and transporters, such as dopamine transporter (DAT) and vesicle monoamine transporter 2 (VMAT2), PET is also a useful tool for studying of changes of the expression and function of brain receptors and transporters during brain development and maturation (Alexander et al., 2017).

It is well-known that there are changes of glucose metabolism that have some inherent connections with the development of brain transporters and ^18^F-FDG PET may be used to evaluate changes of glucose metabolism (Chugani and Phelps, 1986; Goyal and Raichle, 2018). There are continued efforts to explore changes of other metabolic pathways in brain development such as those related to synapse activity and biometals. DAT are protein molecules located in the dopaminergic nerve terminals, which mediate neuronal uptake of dopamine from extracellular space into extravesicular cytoplasmic compartments. [^11^C] 2-β-carbomethoxy-3-β- (4-fluorophenyl)tropane (^11^C-CFT) is a radioligand with high affinity binding to DAT (Brownell et al., 1996). VMAT2 are responsible for packaging and transporting neurotransmitters, such as dopamine, into synaptic vesicles (Eiden and Weihe, 2011). [^18^F]9-fluoropropyl-(+)-dihydrotetrabenazine (^18^F-FP-(+)-DTBZ) can bind to VMAT2 with high affinity. Both DAT and VMAT2 regulate the synaptic concentration of neurotransmitters in the brain (Hall et al., 2014). Expression level of DAT and VMAT2 reflect the density of dopamine terminals in brain regions, which may change during brain development and pathogenesis of brain disorders such as Parkinson’s disease. It was demonstrated that ^11^C-CFT and ^18^F-FP-(+)-DTBZ could be used for quantification of the density of DAT and VMAT2 in brain (Hsiao et al., 2014; Wood, 2014).

Rat adulthood is defined as full sexual maturity in many studies, and the time window is typically described by age and body weight, which lack clear and unambiguous boundaries. Mengler et al. (2014) found clear indications of ongoing developmental changes of cortical thickness, myelination in the rat brain, which point to the time window of maturity within 3 months of age. In this study, to better characterize the brain development of glucose metabolism, DAT and VMAT2, three radiotracers were utilized to observe the development of rat brains in maturation. We recorded the PET signals of these three radiotracers in Wistar rats from 2 to 12 months of age in a longitudinal study. with this multi-tracer approach, we undertook to chart the interrelations of DAT and VMAT2 *in vivo* during the early life of rats, and find a time window of brain maturation.

## Materials and Methods

### Animals and Radiopharmaceuticals

Wistar rats were purchased from Shanghai Laboratory Animal Center (Shanghai, China) and housed in the animal housing facility, with free access to food and drinking water. ^18^F-PF-(+)-DTBZ, ^11^C-CFT, and ^18^F-FDG were prepared in the radiochemistry facility of PET center, Huashan Hospital, Fudan University for clinical use under requirement of GMP. All small animal experiments were conducted according to a protocol approved by Huashan Hospital, Shanghai, China.

### Study Design

**FIGURE 1:**
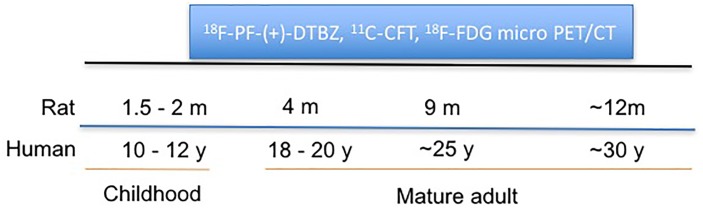
Schematic representation of longitudinal study of change of VMAT2, DAT, and glucose metabolism in rat brains with PET/CT at different ages.

A longitudinal, sequential micro-PET/CT study was performed to assess age-dependent changes of VMAT2, DAT, and glucose metabolism in a group of Wistar rats (male, *n* = 6) from 2 to 12 months of age (equivalent to 12 to 30 years in humans) using ^18^F-PF-(+)-DTBZ, ^11^C-CFT, and ^18^F-FDG, respectively, (Figure 1). Small animal imaging was conducted at 2, 4, 9 months and approximately 12 months of age. Rat ages were defined as the human age equivalents outlined below according to literature (Quinn, 2005).

### Micro-PET/CT Imaging

Micro-PET/CT imaging of Wistar rats was conducted by a method described previously using Inveon microPET-CT scanner (Siemens Inc., United States) (Zilliox et al., 2016). Briefly, the rats were anesthetized using 2–3% isoflurane in medical oxygen (1 L/min) at room temperature with an isoflurane vaporizer (Molecular Imaging Products Company, United States). The rats were positioned in a spread-supine position on the imaging bed and subjected to inhalation of the anesthetic during the PET/CT procedure. Static PET/CT imaging was obtained for 10 min at 60 min post intravenous administration of ^18^F-FDG (∼10 μCi/g body weight), at 60 min post intravenous administration of ^18^F-PF-(+)-DTBZ (∼10 μCi/g body weight), and 45 min post administration of ^11^C-CFT (∼20 μCi/g body weight). The imaging time is based on previous dynamic study we performed. PET/CT images were reconstructed using the ordered subsets expectation maximization 3D algorithm (OSEM3D), and data was reviewed using the Inveon Research Workplace (IRW) software (Siemens) and processed by PMOD software (version 3.4, PMOD Technologies Ltd., Zurich, Switzerland).

### Quantification of Radiotracer Activity in the Brain Regions of Rats

**FIGURE 2:**
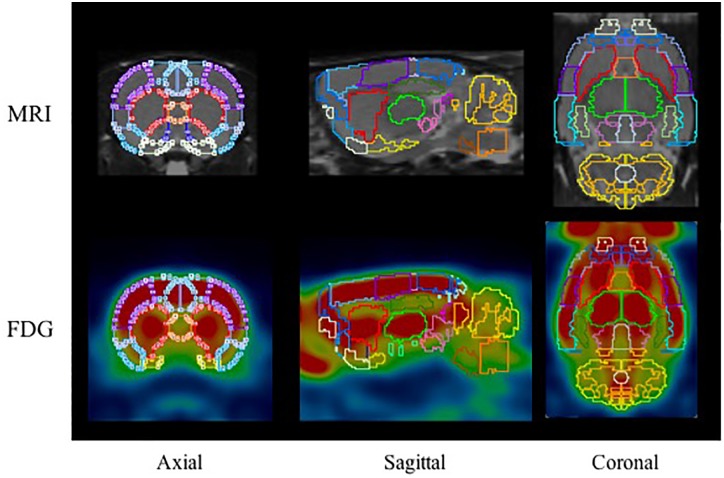
The ROIs applied to the PET images using MR imaging-based atlas by PMOD (top) and corresponding example for FDG (bottom). The main brain regions included: cerebellum (marigold), accumbens (blue), amygdala (yellow), striatum (red), hippocampus posterior (green), midbrain (purple), thalamus (lime), and cortex etc.

^18^F-FDG PET images were automatically fused using the PMOD FDG rat brain template (Schiffer et al., 2006) while ^18^F-PF-(+)-DTBZ and ^11^C-CFT PET images were manually fused with the T2-MRI template. 58 brain regions of interest (ROIs) were collected on the PET/CT images with reference to the MR imaging-based atlas (Schiffer atlas) (Schiffer et al., 2006). The regions analyzed included: cerebellum, accumbens (left and right), amygdala (left and right), striatum (left and right), hippocampus posterior (left and right), hypothalamus (left and right), olfactory (left and right), midbrain (left and right), thalamus (left and right), and cortex etc., All collected brain regions with a sample of ^18^F-FDG are present in Figure 2. The quantity of ^18^F-PF-(+)-DTBZ, ^11^C-CFT, and ^18^F-FDG activity was obtained and recorded as a standardized uptake value (SUV). SUV ratio (SUVR) was calculated by SUV uptake in the specific brain regions compared to that of the cerebellum.

### Statistical Analysis

In order to determine whether the SUV and SUVR of [^18^F]FDG, [^11^C]CFT, [^18^F]DTBZ in these rats differ among different ages, we applied two-way analysis of variance (ANOVA), followed by Tukey multiple comparison post-test, with the between-subjects factor representing the different age groups and the within-subjects factor representing the brain regions. Statistical analyses was performed in Prism (version 7.0, GraphPad Software, San Diego, CA, United States). A *P*-value of less than 0.05 was considered statistically significant.

## Results

### Comparison of ^18^F-PF-(+)-DTBZ, ^11^C-CFT, and ^18^F-FDG Uptake in Rat Brains

**FIGURE 3:**
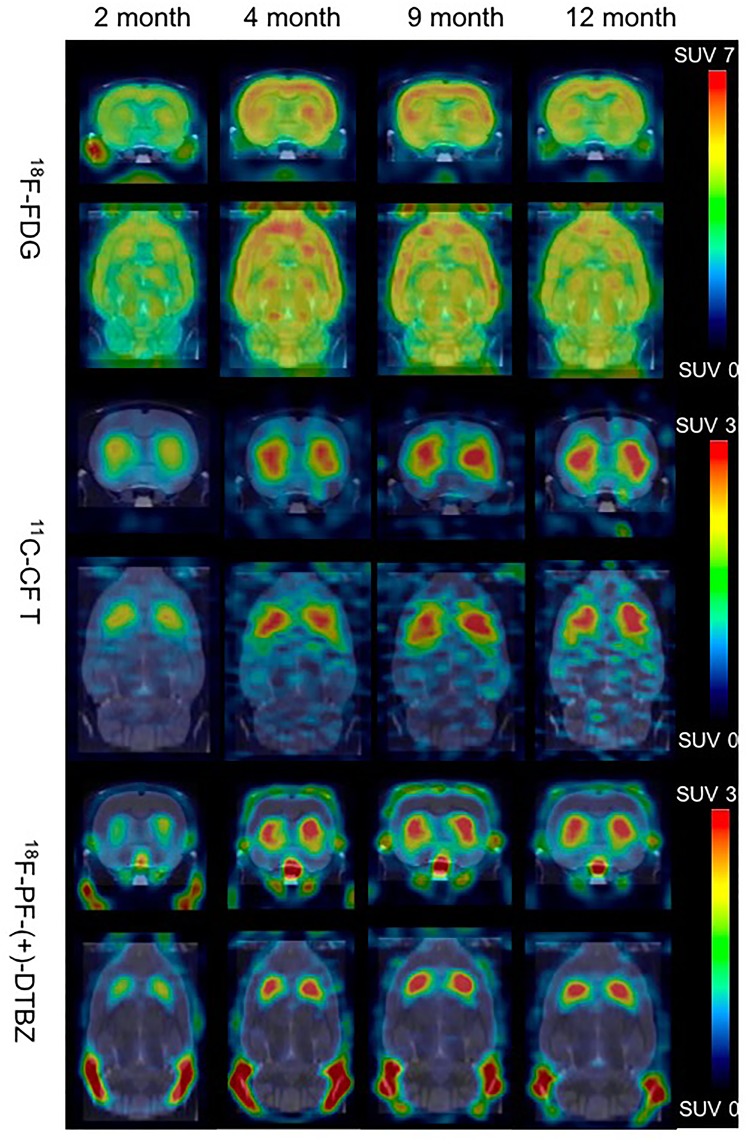
Biodistribution of radiotracer activity in rat brains by PET/CT imaging at different ages, post intravenous administration of ^18^F-PF-(+)-DTBZ, ^11^C-CFT and ^18^F-FDG, respectively. There was increase of brain uptake of these 3 radiotracers increased from 2 months to 4, 9, 12 months of age by PET quantification.

Bodyweight of these rats increased from 222.0 ± 14.6 to 424.5 ± 7.2 from 2 to 12 months of age, and kept in a relatively stable range between 371.7 ± 32.9 and 424.5 ± 7.2 from 4 to 12 months of age (Supplementary Figure S1). ^18^F-PF-(+)-DTBZ and ^11^C-CFT displayed similar distribution patterns in rats after intravenous administration. Both radiotracers mainly accumulated in the striatum and accumbens, yet uptake of ^18^F-PF-(+)-DTBZ could also be found in the skull (Figure 3). The pattern of ^18^F-FDG uptake in rat brains was different from these two tracers, as the cortex, striatum and accumbens had the highest FDG uptake, followed by the cerebellum, while the pituitary gland demonstrated the lowest uptake (Figure 3 and Supplementary Tables S1–S6). Additionally, age-dependent increase of whole brain radiotracer uptake were observed, radioactivity uptake of these 3 radiotracers were higher in the rat brains at 4, 9, 12 months of age than that at 2 months of age.

### Age-Dependent Changes of ^11^C-CFT Uptake in Rat Brains

A decrease in DAT expression was observed in human and small animal brain aging (Hall et al., 2014). In order to determine whether there are age-dependent changes of DAT expression in brain development and maturation, a longitudinal PET/CT study was performed to measure ^11^C-CFT in the brains of rats at 2, 4, 9, and 12 months of age.

**FIGURE 4:**
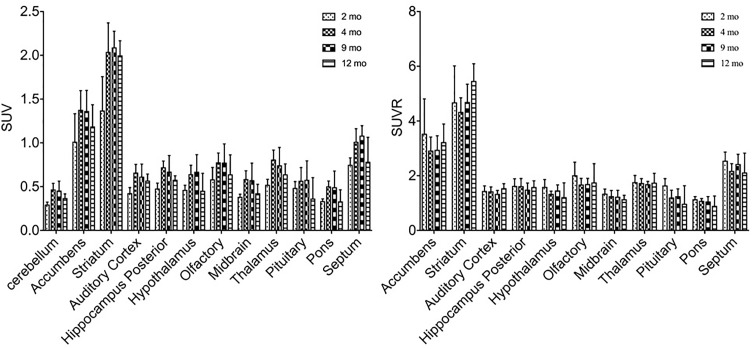
Alterations of SUV and SUVR of ^11^C-CFT in brain regions from 2 to 12 months of age. SUV (left) increased from 2 to 4 months of age in the striatum and accumbens, along with other regions. SUVR (right) did not fluctuate in most brain regions, aside from varying patterns in the striatum and accumbens. All data were presented by mean ±*SD*.

^11^C-CFT uptake (SUV) reached a slightly descending plateau at 4 months of age in the striatum and accumbens (Figure 4). The striatum displayed a significantly lower ^11^C-CFT uptake at 2 months of age (SUV: 1.37 ± 0.39) compared to 4 months (SUV: 2.04 ± 0.33, *P* < 0.0001), 8 months (SUV: 2.09 ± 0.18, *P* < 0.0001 ) and 12 months (SUV: 2.00 ± 0.17, *P* < 0.0001). The same trend was found in the accumbens, as increased SUV was observed from 2 months (1.01 ± 0.32) to 4 months (1.38 ± 0.22, *P* = 0.0021), 9 months (1.36 ± 0.24, *P* = 0.0033), and 12 months (1.19 ± 0.25, *P* = 0.3705). SUV uptake in nearly all other brain regions did not demonstrate significant change, aside from the thalamus from 2 to 4 months (*P* = 0.02) (Supplementary Table S5).

SUVR of ^11^C-CFT displayed a different pattern compared to that of SUV uptake. Striatum SUVR did not change significantly from 2 to 4 months of age (4.68 ± 1.33 to 4.33 ± 0.52), but increased from 2 to 12 months (4.68 ± 1.33 to 5.47 ± 0.63, *P* = 0.04) and also from 4 to 12 months (+26.3%, *P* = 0.0017) (Supplementary Table S6).

### Age-Dependent Changes of ^18^F-PF-(+)-DTBZ Radioactivity in Rat Brain

**FIGURE 5:**
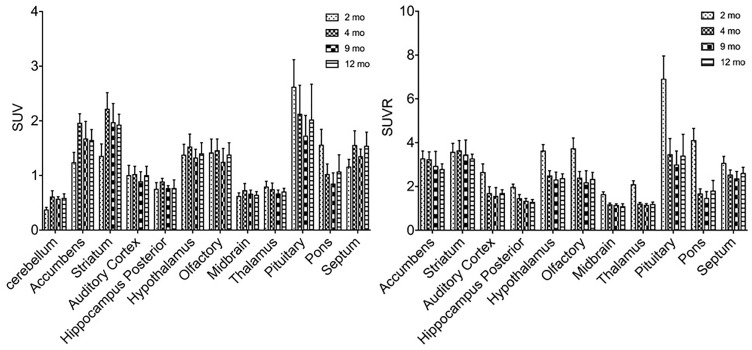
SUV and SUVR of ^18^F-PF-(+)-DTBZ in brain regions of rats at different ages. Increased SUV (left) and decreased SUVR (right) ^18^F-PF-(+)-DTBZ uptake was observed from 2 months of age onward. All data were presented by mean ±*SD*.

Similar alterations of VMAT2 expression were found in these rats by PET imaging using ^18^F-PF-(+)-DTBZ compared to ^11^C-CFT, when quantifying by SUV (Figure 5). Highest ^18^F-PF-(+)-DTBZ activity accumulated in the striatum, accumbens, and septum. Additionally, the striatum displayed significantly low ^18^F-PF-(+)-DTBZ uptake at 2 months of age (SUV: 1.36 ± 0.22) compared to 4 months (SUV: 2.22 ± 0.29, *P* < 0.0001 ), 9 months (SUV: 1.98 ± 0.34, *P* = 0.0005), and 12 months (SUV: 1.93 ± 0.19, *P* = 0.0015) of age. Again, the same pattern in the accumbens that was found using ^11^C-CFT was observed, as there was significantly low ^18^F-PF-(+)-DTBZ uptake at 2 months of age (SUV: 1.24 ± 0.18) compared with 4 months (SUV: 1.96 ± 0.17, *P* < 0.0001), 9 months (SUV: 1.68 ± 0.31, *P* = 0.0273), and 12 months (SUV: 1.65 ± 0.19, *P* = 0.0442) of age. Note that uptake in certain brain regions, such as cortex, were not included here because they were affected by high bone uptake (Supplementary Table S3).

A slight, non-significant decline SUVR of ^18^F-PF-(+)-DTBZ was observed from 2 to 12 months of age at the striatum (-8.4%) and accumbens (-14.7%). Yet, significant decreases of ^18^F-PF-(+)-DTBZ SUVR were observed in other brain regions in this period, such as in the hippocampus (*P* = 0.0463) and auditory cortex (*P* = 0.0021) (Supplementary Table S4).

### Age-Dependent Changes of Glucose Metabolism in Brains of Rats

**FIGURE 6:**
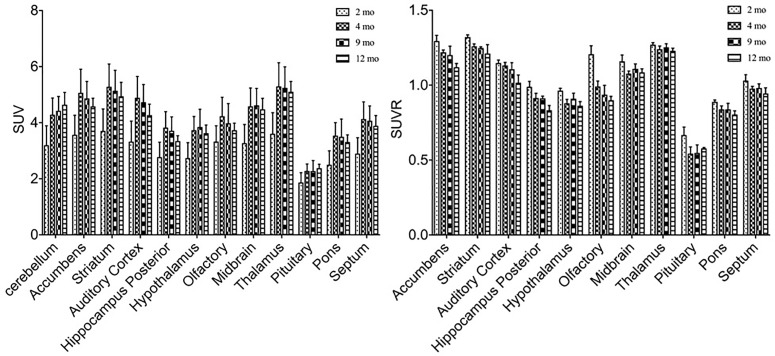
Age-dependent changes of SUV and SUVR uptake of ^18^F-FDG in brain regions of rats at different ages by a longitudinal PET study after intravenous administration. Increased SUV (left) and decreased SUVR (right) of ^18^F-FDG was observed from 2 months of age to older ages. All data were presented by mean ± SD.

In order to study the role of glucose metabolism during brain growth and the relationship among glucose metabolism and DAT and VMAT2 expression, we also examined glucose changes in the brain using ^18^F-FDG (Figure 6). A similar increased age-dependent FDG SUV uptake pattern was discovered from 2 months to 4, 9, and 12 months of age, as seen in both ^11^C-CFT and ^18^F-PF-(+)-DTBZ. However, high accumulations were found in the cortex, striatum, and accumbens, while the lowest uptake was observed in the pituitary gland. SUV in the striatum at 2 months of age increased significantly from 3.71 ± 0.78 to 5.28 ± 0.81 (*P* < 0.0001) at 4 months of age, and then decreased gradually from 4 to 9 and 12 months of age. Almost all of the brain regions demonstrated a significantly lower FDG SUV uptake at 2 months compared to older ages, except in the pituitary gland, which possessed the lowest uptake and displayed no significant change of uptake at different ages. SUVR uptake decreased significantly from 2 months to older ages in most brain regions, including the striatum, accumbens and cortex etc., this decrease was not observed in the thalamus, pons, and auditory cortex (Supplementary Tables S1, S2).

### Relationship Between Age-Dependent Changes of ^18^F-FDG, ^18^F-PF-(+)-DTBZ, and ^11^C-CFT Uptake in Brains

**FIGURE 7:**
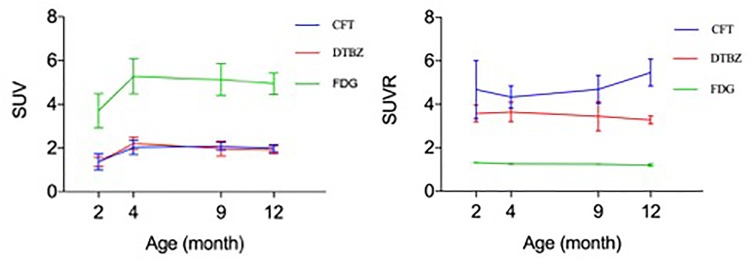
Correlation of ^18^F-FDG, ^18^F-PF-(+)-DTBZ and ^11^C-CFT uptake in the striatum of rats. A similar trend of SUV (left) at different ages for these 3 radiotracers, age-dependent increase uptake was observed from 2 months of age to an older age. And a coincidence of SUV uptake pattern for ^18^F-PF-(+)-DTBZ and ^11^C-CFT, but no correlation was observed for SUVR uptake (right) of these radiotracers. All data were presented by mean ±*SD*.

In order to determine the relationship of glucose metabolism, DAT, and VMAT2 during brain development, we compared the age-dependent change of striatal uptake of these three radiotracers. There existed a similar pattern of age-dependent change in regional brain SUV uptake among these three radiotracers from 2 to 12 month of age, but no correlation of SUVR uptake in these brain regions. As shown in Figure 7, ^18^F-FDG, ^18^F-PF-(+)-DTBZ, and ^11^C-CFT SUV in the striatum displayed similar patterns by increasing from 2 to 4 months and then reaching a slightly descending plateau until 12 months of age. Interestingly, SUV of ^18^F-PF-(+)-DTBZ, and ^11^C-CFT were similar at different individual ages (*P* > 0.05 at 2, 4, 9, and 12 months). However, SUVR demonstrated different patterns in striatum for these three radiotracers. The striatum ^11^C-CFT SUVR was higher than ^18^F-PF-(+)-DTBZ SUVR at all ages.

## Discussion

Investigation of age-dependent changes of various metabolic pathways or expression or function of receptors or transporters is critical for a better understanding regulation of brain development and maturation. Many studies revealed brain function alterations in brain aging, such as fluctuations in DAT, glucose levels, and copper metabolism (Kawamura et al., 2003; Peng et al., 2018). However, few studies were dedicated to study of morphological and functional changes in brain development and maturation at adolescent population, partly due to technical limitation and other factors such as access to adolescent human subject population. *In vivo* imaging techniques, such as MRI and PET, are useful tools for monitoring disease progression along with changes in biological pathways and metabolism. The definition of brain maturity is divided into many types, such as social, cognitive, and neurotransmitter system etc., Previous studies using MRI found brain structures, especially the rat brain cortex, are not fully developed at 3 months of age. Therefore, they recommend careful examination of the brain structures before longitudinal examinations (Mengler et al., 2014). However, the function examination of brain development is rarely considered, particularly the examination of expression of dopamine-related receptors and transporters in brain maturation.

Dopamine levels are considered to represent the density of dopamine terminals. DAT and VMAT2 can reflect dopamine levels, and they are biomarkers brain disease and used to study brain aging (Colebrooke et al., 2006; Troiano et al., 2010; Oh et al., 2012). DAT and VMAT2 in the striatum were found to be decreased in the brains of aging rats and in human brains using ^11^C-CFT and ^18^F-PF-(+)-DTBZ as radiotracers (Kawamura et al., 2003; Hall et al., 2014). More importantly, DAT and VMAT2 were biomarkers for Parkinson’s Disease (PD). ^11^C-CFT and ^18^F-PF-(+)-DTBZ were applied to diagnose PD and evaluate the severity of PD (Nurmi et al., 2000; Hsiao et al., 2014). The increased expression of dopamine terminals can reflect the process of brain maturation (O’Donnell, 2010). Age-dependent changes of the DAT and VMAT2 biomarkers could be useful in the assessment of density changes of dopamine terminals associated with maturation.

In this study, we found age-dependent increases in FDG uptake in rat brains from 2 to 4 months of age, and a slightly non-significant decline of glucose metabolism was observed from 4 to 12 month. This finding is different from decreased glucose levels in aging brains described in previous reports (Chetelat et al., 2013; Ewers et al., 2014; Nugent et al., 2014). The increased glucose metabolism may be caused by the increase in glucose demand in brain maturation. In addition to the increase in glucose metabolism, expression of DAT and VMAT2 was also elevated from 2 to 4 months of age within the striatum. This increased density of dopamine terminals in the striatum and suggests an increase in dopamine level or concentration. Mechanisms of concurrent increase of FDG uptake and elevated expression of DAT and VMAT2 are not clear.

Aside from the measurement of SUV, we also calculated the SUVR of these three radiotracers. Calculation of SUVR is considered standard practice in clinical ^18^F-FDG PET brain imaging. Interestingly, the pattern of SUVR is different from the pattern of SUV among these three radiotracers. SUV of ^18^F-FDG increased from 2 to 4 months of age in most brain regions, while SUVR declined slightly, which is caused by global increased glucose uptake in rat brains and faster increase of FDG uptake in the cerebellum. For SUVR of ^11^C-CFT and ^18^F-PF-(+)-DTBZ, no significant changes were observed in these brain regions, except in the stratum (2 m vs. 12 m, *P* = 0.04; 4 m vs. 12 m, *P* = 0.002) for ^11^C-CFT. This may be induced by the global brain increase of ^11^C-CFT uptake. The age-dependent higher and increased SUVR of ^11^C-CFT in the striatum compared with a decline in SUVR of ^18^F-PF-(+)-DTBZ showed the potential of ^11^C-CFT in the diagnosis of brain disease in the striatum, such as PD (Yagi et al., 2010).

Owing to the high concentrations of dopamine in the striatal regions, several studies of dopamine in aging have been focused on this region. Different from those studies in aged animals, we found a correlation in the SUV uptake curves of ^11^C-CFT and ^18^F-PF-(+)-DTBZ in the striatum (Figure 7). Both radiotracers could reflect the expression of dopamine levels. Our results indicated the positive correlation of absolute quantification (SUV) of DAT and VMAT2 in the striatum in maturation, which demonstrates growth of dopamine terminals in the striatum, and the numbers of DAT and VMAT2 may also be similar within the striatum. ^18^F-FDG possessed a higher SUV uptake and similar SUV pattern compared with the other two radiotracers within the stratum, indicating the significance of glucose metabolism in the development of DAT and VMAT2 in this region.

The age of small animals is a key impact factor in the study of brain disease, yet many brain studies are based on the assumption of matured brains. When the animals are too young with undeveloped brain-blood barrier (BBB), radiotracers will not need to penetrate the BBB to induce high brain uptake (Patel and Gibson, 2008). Additionally, immature brains will introduce deviation in longitudinal studies. Therefore, mature mice or rats are essential in the evaluation of new radiotracers targeting the brain and longitudinal studies examining brain disease and brain functions. However, about 300 g of body weight or 2 to 3 months of age have often been applied to define the time window of maturity in rats.

In previous studies, volume change, cortical thickness, and cell density in the striatum were found to be stabilized in 3 months of age, but myelination in the striata continued up to 3 months of age and in the cortex up to 6 months (Mengler et al., 2014). Our study confirms that brain synaptic development is not stabilized within the first 4 months, as the densities of DAT and VMAT2 continue grow at 4 months of age, which coincided with previous study. Therefore, 4 months of age may be considered as the window time for brain maturation of dopamine. Longitudinal study for brain, especially PET imaging study, should not start before 4 months of age in rats. And future studies need to better define the end of the developmental phase before a related study can begin.

There were several limitations in this study. Brain size could be influent factor for the measurement of brain uptake. In previous study, brain size did not change significantly after 2 month of age in Wistar rats (Mengler et al., 2014). Brain uptake pattern of these rats at 2 and 12 month matched PMOD template (Supplementary Figure S2), which means brain uptake from PMOD can be used for comparison. That conclusion supported us to start our study from 2 month of age. However, Lack of MRI study for brain size still limit this study. Perfusion of the brain uptake as a consequence of brain development and differential response to anesthetization is another limitation. Different type of anesthetization brought different response to brain uptake. Brain uptake of ^18^F-FDG decreased in condition of isoflurane and ketamine/xylazine anesthetization, no change of striatal ^18^F-PF-(+)-DTBZ uptake was observed in isoflurane but ketamine increased ^18^F-PF-(+)-DTBZ striatal uptake (Toyama et al., 2004; Chen et al., 2016). All of these results indicated anesthetization is one of the confounding factor to brain uptake. Especially at different age of rats, differential response to anesthetization may cause different perfusion pattern, relative proportion of the tracer in the brain may alter. This is also one of the main limitation in this study. All of these limitations desired further study to investigate the infections.

## Conclusion

Age-dependent increase of striatal ^18^F-FDG, ^18^F-PF-(+)-DTBZ and ^11^C-CFT radiotracer uptake was detected in Wistar rats from 2 to 4 months of age, reaching plateau of uptake in matured rats at 4 month of age. The findings suggest that 4 months of age may represent a desirable time point for conducting neurological study of brain disease to avoid confounding effects of ongoing changes of glucose metabolism and neuroreceptor expression on neurological studies involving use of PET and radiopharmaceuticals. The findings support further investigation of age-dependent changes of DAT and VMAT2 expression during brain development and maturation with PET using ^18^F-PF-(+)-DTBZ and ^11^C-CFT radiotracer, respectively.

## Author Contributions

DJ and FX designed and performed the experiments. XL and FX produced the radiotracers. DJ, ZL, FH, CZ, YG, and FX contributed to conception and designed the study. DJ, FX, and FH contributed to analysis and interpretation of data. FX wrote the draft of this manuscript. DJ, NR, FP, and FX contributed to critical review and revision of the manuscript for this article.

## Conflict of Interest Statement

The authors declare that the research was conducted in the absence of any commercial or financial relationships that could be construed as a potential conflict of interest.

## References

[B1] Alexander P. K. Lie Y. Jones G. Sivaratnam C. Bozinvski S. Mulligan R. S. (2017). Management impact of imaging brain vesicular monoamine transporter type 2 in clinically uncertain parkinsonian syndrome with 18F-AV133 and PET. *J. Nucl. Med.* 58 1815–1820. 10.2967/jnumed.116.189019 28490469

[B2] Aljabar P. Bhatia K. K. Murgasova M. Hajnal J. V. Boardman J. P. Srinivasan L. (2008). Assessment of brain growth in early childhood using deformation-based morphometry. *Neuroimage* 39 348–58. 10.1016/j.neuroimage.2007.07.067 17919930

[B3] Brownell A. L. Elmaleh D. R. Meltzer P. C. Shoup T. M. Brownell G. L. Fischman A. J. (1996). Cocaine congeners as PET imaging probes for dopamine terminals. *J. Nucl. Med.* 37 1186–1192. 8965196

[B4] Chen Z. Tang J. Liu C. Li X. Huang H. Xu X. (2016). Effects of anesthetics on vesicular monoamine transporter type 2 binding to 18F-FP-(+)-DTBZ: a biodistribution study in rat brain. *Nucl. Med. Biol.* 43 124–129. 10.1016/j.nucmedbio.2015.09.009 26526872

[B5] Chetelat G. Landeau B. Salmon E. Yakushev I. Bahri M. A. Mezenge F. (2013). Relationships between brain metabolism decrease in normal aging and changes in structural and functional connectivity. *Neuroimage* 76 167–177. 10.1016/j.neuroimage.2013.03.009 23518010

[B6] Choi H. Choi Y. Kim K. W. Kang H. Hwang D. W. Kim E. E. (2015). Maturation of metabolic connectivity of the adolescent rat brain. *eLife* 4:e11571. 10.7554/eLife.11571 26613413 PMC4718811

[B7] Chugani H. T. (1998). A critical period of brain development: studies of cerebral glucose utilization with PET. *Prev. Med.* 27 184–188. 10.1006/pmed.1998.0274 9578992

[B8] Chugani H. T. Phelps M. E. (1986). Maturational changes in cerebral function in infants determined by 18FDG positron emission tomography. *Science* 231 840–843. 10.1126/science.3945811 3945811

[B9] Colebrooke R. E. Humby T. Lynch P. J. McGowan D. P. Xia J. Emson P. C. (2006). Age-related decline in striatal dopamine content and motor performance occurs in the absence of nigral cell loss in a genetic mouse model of Parkinson’s disease. *Eur. J. Neurosci.* 24 2622–2630. 10.1111/j.1460-9568.2006.05143.x 17100850

[B10] Eiden L. E. Weihe E. (2011). VMAT2: a dynamic regulator of brain monoaminergic neuronal function interacting with drugs of abuse. *Ann. N. Y. Acad. Sci.* 1216 86–98. 10.1111/j.1749-6632.2010.05906.x 21272013 PMC4183197

[B11] Ewers M. Brendel M. Rizk-Jackson A. Rominger A. Bartenstein P. Schuff N. (2014). Reduced FDG-PET brain metabolism and executive function predict clinical progression in elderly healthy subjects. *Neuroimage Clin.* 4 45–52. 10.1016/j.nicl.2013.10.018 24286024 PMC3841292

[B12] Goyal M. S. Raichle M. E. (2018). Glucose requirements of the developing human brain. *J. Pediatr. Gastroenterol. Nutr.* 66(Suppl. 3), S46–S49. 10.1097/mpg.0000000000001875 29762377 PMC5959031

[B13] Hall F. S. Itokawa K. Schmitt A. Moessner R. Sora I. Lesch K. P. (2014). Decreased vesicular monoamine transporter 2 (VMAT2) and dopamine transporter (DAT) function in knockout mice affects aging of dopaminergic systems. *Neuropharmacology* 76 146–155. 10.1016/j.neuropharm.2013.07.031 23978383 PMC3811113

[B14] Hsiao I. T. Weng Y. H. Hsieh C. J. Lin W. Y. Wey S. P. Kung M. P. (2014). Correlation of parkinson disease severity and 18F-DTBZ positron emission tomography. *JAMA Neurol.* 71 758–766. 10.1001/jamaneurol.2014.290 24756323

[B15] Kawamura K. Oda K. Ishiwata K. (2003). Age-related changes of the [11C]CFT binding to the striatal dopamine transporters in the Fischer 344 rats: a PET study. *Ann. Nucl. Med.* 17 249–253. 12846549 10.1007/BF02990030

[B16] Khundrakpam B. S. Lewis J. D. Zhao L. Chouinard-Decorte F. Evans A. C. (2016). Brain connectivity in normally developing children and adolescents. *Neuroimage* 134 192–203. 10.1016/j.neuroimage.2016.03.062 27054487

[B17] Mengler L. Khmelinskii A. Diedenhofen M. Po C. Staring M. Lelieveldt B. P. (2014). Brain maturation of the adolescent rat cortex and striatum: changes in volume and myelination. *Neuroimage* 84 35–44. 10.1016/j.neuroimage.2013.08.034 23994458

[B18] Mosconi L. Tsui W. H. Herholz K. Pupi A. Drzezga A. Lucignani G. (2008). Multicenter standardized 18F-FDG PET diagnosis of mild cognitive impairment, Alzheimer’s disease, and other dementias. *J. Nucl. Med.* 49 390–8. 10.2967/jnumed.107.045385 18287270 PMC3703818

[B19] Nugent S. Tremblay S. Chen K. W. Ayutyanont N. Roontiva A. Castellano C. A. (2014). Brain glucose and acetoacetate metabolism: a comparison of young and older adults. *Neurobiol. Aging* 35 1386–1395. 10.1016/j.neurobiolaging.2013.11.027 24388785

[B20] Nurmi E. Ruottinen H. M. Kaasinen V. Bergman J. Haaparanta M. Solin O. (2000). Progression in Parkinson’s disease: a positron emission tomography study with a dopamine transporter ligand [18F]CFT. *Ann. Neurol.* 47 804–808. 10852547

[B21] O’Donnell P. (2010). Adolescent maturation of cortical dopamine. *Neurotox. Res.* 18 306–312. 10.1007/s12640-010-9157-9153 20151241

[B22] Oh M. Kim J. S. Kim J. Y. Shin K. H. Park S. H. Kim H. O. (2012). Subregional patterns of preferential striatal dopamine transporter loss differ in Parkinson disease, progressive supranuclear palsy, and multiple-system atrophy. *J. Nucl. Med.* 53 399–406. 10.2967/jnumed.111.095224 22323779

[B23] Patel S. Gibson R. (2008). *In vivo* site-directed radiotracers: a mini-review. *Nucl. Med. Biol.* 35 805–815. 10.1016/j.nucmedbio.2008.10.002 19026942

[B24] Peng F. Xie F. Muzik O. (2018). Alteration of copper fluxes in brain aging: a longitudinal study in rodent using 64CuCl2-PET/CT. *Aging Dis.* 9 109–118. 10.14336/ad.2017.1025 29392086 PMC5772849

[B25] Quinn R. (2005). Comparing rat’s to human’s age: how old is my rat in people years? *Nutrition* 21 775–777. 10.1016/j.nut.2005.04.002 15925305

[B26] Schiffer W. K. Mirrione M. M. Biegon A. Alexoff D. L. Patel V. Dewey S. L. (2006). Serial microPET measures of the metabolic reaction to a microdialysis probe implant. *J. Neurosci. Methods* 155 272–284. 10.1016/j.jneumeth.2006.01.027 16519945

[B27] Smailagic N. Vacante M. Hyde C. Martin S. Ukoumunne O. Sachpekidis C. (2015). 18F-FDG PET for the early diagnosis of Alzheimer’s disease dementia and other dementias in people with mild cognitive impairment (MCI). *Cochrane Database Syst. Rev.* 1:CD010632. 10.1002/14651858.CD010632.pub2 25629415 PMC7081123

[B28] Sowell E. R. Thompson P. M. Leonard C. M. Welcome S. E. Kan E. Toga A. W. (2004). Longitudinal mapping of cortical thickness and brain growth in normal children. *J. Neurosci.* 24 8223–8231. 10.1523/jneurosci.1798-04.2004 15385605 PMC6729679

[B29] Toyama H. Ichise M. Liow J. S. Vines D. C. Seneca N. M. Modell K. J. (2004). Evaluation of anesthesia effects on [18F]FDG uptake in mouse brain and heart using small animal PET. *Nucl. Med. Biol.* 31 251–256. 10.1016/s0969-8051(03)00124-120 15013491

[B30] Troiano A. R. Schulzer M. de la Fuente-Fernandez R. Mak E. McKenzie J. Sossi V. (2010). Dopamine transporter PET in normal aging: dopamine transporter decline and its possible role in preservation of motor function. *Synapse* 64 146–151. 10.1002/syn.20708 19852071

[B31] Wood H. (2014). Parkinson disease: 18F-DTBZ PET tracks dopaminergic degeneration in patients with Parkinson disease. *Nat. Rev. Neurol.* 10:305. 10.1038/nrneurol.2014.81 24840973

[B32] Yagi S. Yoshikawa E. Futatsubashi M. Yokokura M. Yoshihara Y. Torizuka T. (2010). Progression from unilateral to bilateral parkinsonism in early parkinson disease: implication of mesocortical dopamine dysfunction by PET. *J. Nucl. Med.* 51 1250–1257. 10.2967/jnumed.110.076802 20660377

[B33] Zilliox L. A. Chadrasekaran K. Kwan J. Y. Russell J. W. (2016). Diabetes and cognitive impairment. *Curr. Diab. Rep.* 16:87. 10.1007/s11892-016-0775-x 27491830 PMC5528145

