# Supplementary material for: Increased Vesicular Monoamine Transporter 2 (VMAT2) and Dopamine Transporter (DAT) Expression in Adolescent Brain Development: A Longitudinal Micro-PET/CT Study in Rodent
Source: Front Neurosci. 2019 Jan 15;12:1052. doi: 10.3389/fnins.2018.01052 (PMC6340981; doi:10.3389/fnins.2018.01052)
Supplement: Supplementary file 1 [file Data_Sheet_1.PDF]

Supplement material

Increased vesicular monoamine transporter 2 (VMAT2) and dopamine transporter (DAT) expression in adolescent brain development: A longitudinal Micro-PET/CT study in rodent

Donglang Jiang[1], Xiuhong Lu[1], Zijing Li[2], Nicklas Rydberg[3], Chuantao Zuo[1], Fangyu Peng[3], Fengchun Hua*[1], Yihui Guan*[1], Fang Xie*[1]

1. PET Center, Huashan Hospital, Fudan University, 200040, Shanghai, China
2. Center for Molecular Imaging and Translational Medicine, State Key Laboratory of Molecular Vaccinology and Molecular Diagnostics, School of Public Health, Xiamen University, 361102, Xiamen, Fujian, China
3. Department of Radiology, University of Texas Southwestern Medical Center, 75390, Dallas, TX, USA

* Correspondence:

Dr. Fang Xie
fangxie@fudan.edu.cn
Dr. Yihui Guan
guanyihui@hotmail.com
Dr. Fengchun Hua
huafc@hotmail.com

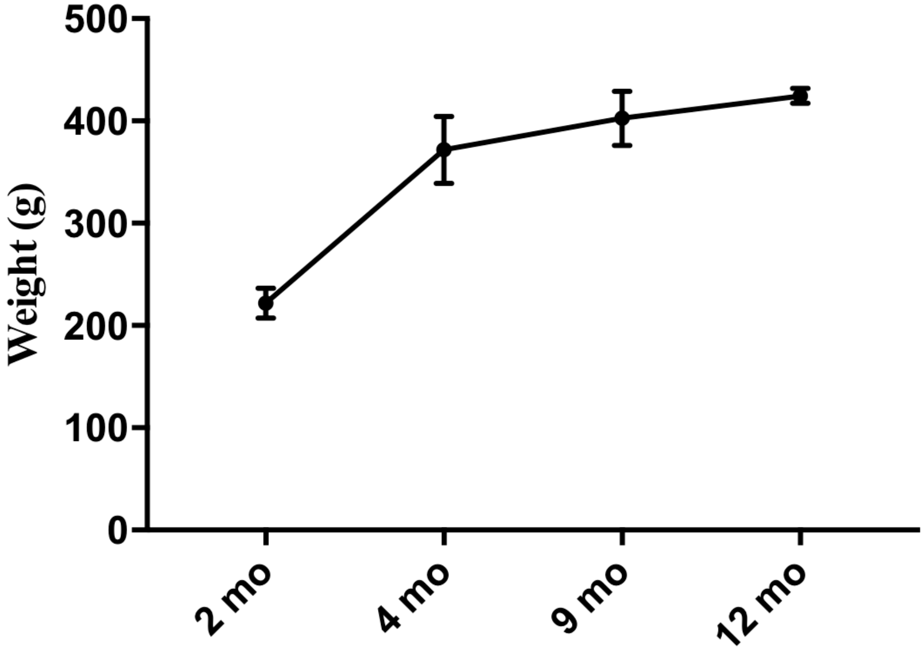

Figure S1: Change of body weight from 2 to 12 months of age.

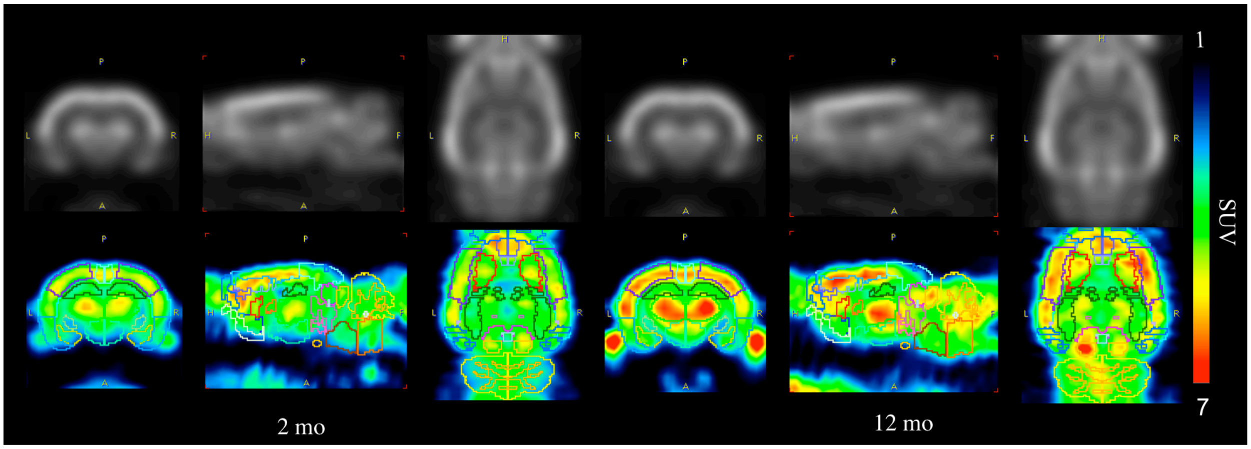

Fig S2: Comparison of FDG uptake pattern at age of 2 months and 12 months at the same rat. FDG uptake pattern did not alter much in PMOD brain regions template (down, brain regions were same as Fig.2) at these 2 ages.

Table S1. Regional SUV of ^{18}F-FDG at Different Ages

	2 mo	4 mo	9 mo	12 mo
cerebellum	3.20 ± 0.70	4.29 ± 0.59	4.42 ± 0.51	4.64 ± 0.44
Accumbens	3.57 ± 0.70	5.07 ± 0.84	4.87 ± 0.60	4.58 ± 0.29
Striatum	3.71 ± 0.78	5.28 ± 0.81	5.14 ± 0.73	4.94 ± 0.50
Auditory Cortex	3.32 ± 0.73	4.89 ± 0.76	4.73± 0.63	4.27 ± 0.39
Hippocampus Posterior	2.77 ± 0.53	3.82 ± 0.57	3.71 ± 0.50	3.35 ± 0.19
Hypothalamus	2.74 ± 0.55	3.73 ± 0.49	3.85 ± 0.64	3.62 ± 0.29
Olfactory	3.33 ± 0.56	4.23 ± 0.67	3.98 ± 0.70	3.73 ± 0.25
Midbrain	3.27 ± 0.67	4.58 ± 0.65	4.62 ± 0.60	4.48 ± 0.39
Thalamus	3.61 ± 0.75	5.29 ± 0.84	5.25 ± 0.75	5.10 ± 0.37
Pituitary	1.87 ± 0.35	2.28 ± 0.24	2.28 ± 0.38	2.38 ± 0.14
Pons	2.50 ± 0.50	3.54 ± 0.47	3.50 ± 0.63	3.31 ± 0.25
Septum	2.90 ± 0.57	4.13 ± 0.61	4.07 ± 0.53	3.89 ± 0.36

All data were presented by mean ± SD

Table S2. Regional SUVR of ^{18}F-FDG at Different Ages

	2 mo	4 mo	9 mo	12 mo
Accumbens	1.29 ± 0.04	1.22 ± 0.01	1.20 ± 0.06	1.12 ± 0.02
Striatum	1.32 ± 0.01	1.26 ± 0.02	1.25 ± 0.01	1.21 ± 0.06
Auditory Cortex	1.15 ± 0.02	1.13 ± 0.02	1.11 ± 0.04	1.02 ± 0.05
Hippocampus Posterior	0.99 ± 0.04	0.92 ± 0.03	0.91 ± 0.01	0.83 ± 0.03
Hypothalamus	0.96 ±0.02	0.88 ± 0.03	0.91 ± 0.04	0.86 ± 0.03
Olfactory	1.21 ± 0.06	0.99 ± 0.04	0.94 ± 0.06	0.90 ± 0.03
Midbrain	1.16 ± 0.04	1.08 ± 0.02	1.11 ± 0.03	1.09 ± 0.02
Thalamus	1.27 ± 0.01	1.24 ± 0.02	1.25 ± 0.02	1.23 ± 0.02
Pituitary	0.67 ± 0.05	0.54 ± 0.04	0.55 ± 0.05	0.58 ± 0.01
Pons	0.89 ± 0.01	0.84 ± 0.02	0.84 ± 0.04	0.81 ± 0.02
Septum	1.03 ± 0.04	0.98 ± 0.01	0.98 ± 0.03	0.95 ± 0.04

All data were presented by mean ± SD

Table S3. Regional SUV of ^{18}F-PF-(+)-DTBZ at Different Ages

	2 mo	4 mo	9 mo	12 mo
cerebellum	0.38 ± 0.04	0.62 ± 0.11	0.57 ± 0.04	0.59 ± 0.07
Accumbens	1.24 ± 0.18	1.96 ± 0.17	1.68 ± 0.31	1.65 ± 0.19
Striatum	1.36 ± 0.22	2.22 ± 0.29	1.98 ± 0.34	1.93 ± 0.19
Auditory Cortex	1.01 ± 0.18	1.03 ± 0.14	0.89 ± 0.18	1.01 ± 0.16
Hippocampus Posterior	0.76 ± 0.11	0.89 ± 0.06	0.77 ± 0.04	0.77 ± 0.15
Hypothalamus	1.38 ± 0.19	1.53 ± 0.23	1.33 ± 0.15	1.40 ± 0.20
Olfactory	1.42 ± 0.24	1.46 ± 0.20	1.25 ± 0.25	1.38 ± 0.21
Midbrain	0.62 ± 0.06	0.73 ± 0.12	0.67 ± 0.07	0.65 ± 0.06
Thalamus	0.80 ± 0.10	0.75 ± 0.11	0.67 ± 0.06	0.71 ± 0.06
Pituitary	2.62 ± 0.49	2.13 ± 0.52	1.73 ± 0.37	2.02 ± 0.65
Pons	1.56 ± 0.28	1.03 ± 0.18	0.85 ± 0.20	1.07 ± 0.31
Septum	1.16 ± 0.13	1.56 ± 0.26	1.35 ± 0.13	1.54 ± 0.25

All data were presented by mean ± SD

Table S4. Regional SUVR of ^{18}F-PF-(+)-DTBZ at Different Ages

	2 mo	4 mo	9 mo	12 mo
Accumbens	3.28 ± 0.34	3.24 ± 0.35	2.94 ± 0.67	2.80 ± 0.23
Striatum	3.58 ± 0.39	3.65 ± 0.44	3.46 ± 0.67	3.28 ± 0.19
Auditory Cortex	2.66 ± 0.38	1.69 ± 0.30	1.57 ± 0.39	1.70 ± 0.15
Hippocampus Posterior	1.99 ± 0.12	1.47 ± 0.16	1.34 ± 0.11	1.30 ± 0.11
Hypothalamus	3.64 ± 0.27	2.50 ± 0.22	2.32 ± 0.34	2.38 ± 0.20
Olfactory	3.75 ± 0.46	2.40 ± 0.28	2.19 ± 0.53	2.35 ± 0.30
Midbrain	1.65 ± 0.10	1.19 ± 0.06	1.16 ± 0.06	1.11 ± 0.11
Thalamus	2.10 ± 0.16	1.22 ± 0.06	1.17 ± 0.05	1.20 ± 0.09
Pituitary	6.92 ± 1.04	3.47 ± 0.71	3.01 ± 0.61	3.41 ± 0.98
Pons	4.12 ± 0.54	1.68 ± 0.22	1.48 ± 0.29	1.81 ± 0.47
Septum	3.08 ± 0.29	2.54 ± 0.21	2.37 ± 0.32	2.61 ± 0.27

All data were presented by mean ± SD

Table S5. Regional SUV of ^{11}C-CFT at Different Ages

	2 mo	4 mo	9 mo	12 mo
cerebellum	0.30 ± 0.03	0.47 ± 0.07	0.46 ± 0.11	0.37 ± 0.05
Accumbens	1.01 ± 0.32	1.38 ± 0.22	1.36 ± 0.24	1.19 ± 0.25
Striatum	1.37 ± 0.39	2.04 ± 0.33	2.09 ± 0.18	2.00 ± 0.17
Auditory Cortex	0.43 ± 0.06	0.66 ± 0.09	0.61 ± 0.14	0.57 ± 0.07
Hippocampus Posterior	0.48 ± 0.06	0.72 ± 0.07	0.67 ± 0.18	0.58 ± 0.04
Hypothalamus	0.46 ± 0.05	0.64 ± 0.10	0.67 ± 0.20	0.45 ± 0.20
Olfactory	0.58 ± 0.14	0.78 ± 0.11	0.78 ± 0.21	0.64 ± 0.22
Midbrain	0.38 ± 0.03	0.59 ± 0.09	0.57 ± 0.19	0.42 ± 0.10
Thalamus	0.52 ± 0.06	0.81 ± 0.11	0.74 ± 0.20	0.64 ± 0.12
Pituitary	0.48 ± 0.07	0.57 ± 0.15	0.58 ± 0.22	0.36 ± 0.24
Pons	0.34 ± 0.03	0.50 ± 0.06	0.49 ± 0.18	0.33 ± 0.13
Septum	0.75 ± 0.08	1.01 ± 0.15	1.08 ± 0.11	0.78 ± 0.28

All data were presented by mean ± SD

Table S6. Regional SUVR of ^{11}C-CFT at Different Ages

	2 mo	4 mo	9 mo	12 mo
Accumbens	3.53 ±1.27	2.92 ± 0.49	2.95 ± 0.51	3.23 ± 0.66
Striatum	4.68 ± 1.33	4.33 ± 0.52	4.69 ± 0.65	5.47 ± 0.63
Auditory Cortex	1.44 ± 0.19	1.43 ± 0.16	1.34 ± 0.13	1.55 ± 0.16
Hippocampus Posterior	1.63 ± 0.26	1.62 ± 0.28	1.49 ± 0.24	1.59 ± 0.23
Hypothalamus	1.59 ±0.27	1.34 ± 0.10	1.46 ± 0.20	1.22 ± 0.52
Olfactory	2.02 ± 0.48	1.68 ± 0.22	1.69 ± 0.21	1.76 ± 0.68
Midbrain	1.34 ± 0.17	1.25 ± 0.21	1.24 ± 0.23	1.14 ± 0.14
Thalamus	1.77 ± 0.24	1.73 ± 0.16	1.69 ± 0.10	1.75 ± 0.34
Pituitary	1.65 ± 0.25	1.21 ± 0.26	1.25 ± 0.27	0.98 ± 0.66
Pons	1.14 ± 0.10	1.08 ± 0.08	1.06 ± 0.18	0.90 ± 0.36
Septum	2.55 ± 0.32	2.18 ± 0.26	2.43 ± 0.35	2.12 ± 0.71

All data were presented by mean ± SD
